# Epstein-Barr virus induced hyperbilirubinemia and jaundice: a case report

**DOI:** 10.1186/s12879-026-12761-5

**Published:** 2026-07-22

**Authors:** Carly Perkowski, Rachel Cox, Kelsey Wilson

**Affiliations:** 1 Wright State University Boonshoft School of Medicine, 3640 Colonel Glenn Hwy, Dayton, OH 45435 USA; 2 Department of Internal Medicine, Wright State University Boonshoft School of Medicine, Dayton, OH USA; 3 Wright-Patterson Medical Center, Dayton, OH USA

**Keywords:** Epstein-Barr virus, Infectious mononucleosis, Hyperbilirubinemia, Autoimmune hemolytic anemia (AIHA), Direct antiglobulin test, Intrahepatic cholestasis

## Abstract

**Background:**

Ebstein-Barr virus (EBV) is the primary cause of infectious mononucleosis (IM), which typically presents with fever, sore throat, and malaise, and is often associated with mild elevations in liver enzymes.

**Case presentation:**

We present the case of a 30-year-old postpartum Caucasian female who developed painless jaundice. Laboratory evaluation revealed markedly elevated total and direct bilirubin, anemia, and exam showed splenomegaly. Serologic testing confirmed acute EBV infection and additional laboratory findings suggested a hemolytic process, including positive direct antiglobulin test and reticulocytosis. Her symptoms and liver function abnormalities gradually improved with supportive care, underscoring EBV as a rare cause of significant hyperbilirubinemia in adults.

**Conclusion:**

This case highlights the need to maintain a broad differential when evaluating hyperbilirubinemia, including atypical viral etiologies such as Epstein-Barr virus.

## Introduction

Epstein–Barr virus (EBV), a ubiquitous herpesvirus, is the primary cause of infectious mononucleosis (IM), commonly affecting adolescents and young adults aged 15 to 25 years [1]​. IM typically presents with malaise, fever, sore throat, lymphadenopathy and hepatosplenomegaly with symptoms usually resolving spontaneously within a month [1]​. While transaminase elevations are observed in approximately 80% of IM cases, jaundice is relatively rare, occurring in only 5–6.6% of patients ​ [2]​. Total bilirubin levels exceeding 6 mg/dL are seen in fewer than 5% of cases, and paradoxically, elevated bilirubin has been found to inversely correlate with typical IM presentations ​ [3, 4]​. Though EBV-related hepatitis is usually mild and self-limited, in rare cases, it may progress to hepatic failure with severe jaundice ​ [5]​. We report a case of EBV-induced hyperbilirubinemia in a patient with minimal constitutional symptoms, underscoring the need to consider EBV in the differential diagnosis of hepatocellular–cholestatic liver injury with jaundice, even when classic mononucleosis features are absent.

This case was previously presented as a meeting abstract at the 2023 Tri-Service Chapters Annual Scientific Meeting on November 8, 2023.

## Case description

A 30-year-old Caucasian woman, five-months postpartum, presented to the emergency department with two days of painless jaundice following recent treatment for a urinary tract infection (UTI) with cefuroxime three days prior to presentation. She reported only mild fatigue, had been compliant with antibiotics, and noted resolution of UTI symptoms. She denied recent travel, intravenous drug use or family history of liver disease. Her history included monogamy with her husband, nightly consumption of one glass of wine, and daily use of a prenatal vitamin.

On presentation, the patient’s vital signs were unremarkable. The physical exam was remarkable for scleral icterus, jaundice, and a soft, nontender abdomen with splenomegaly.

**Fig. 1:**
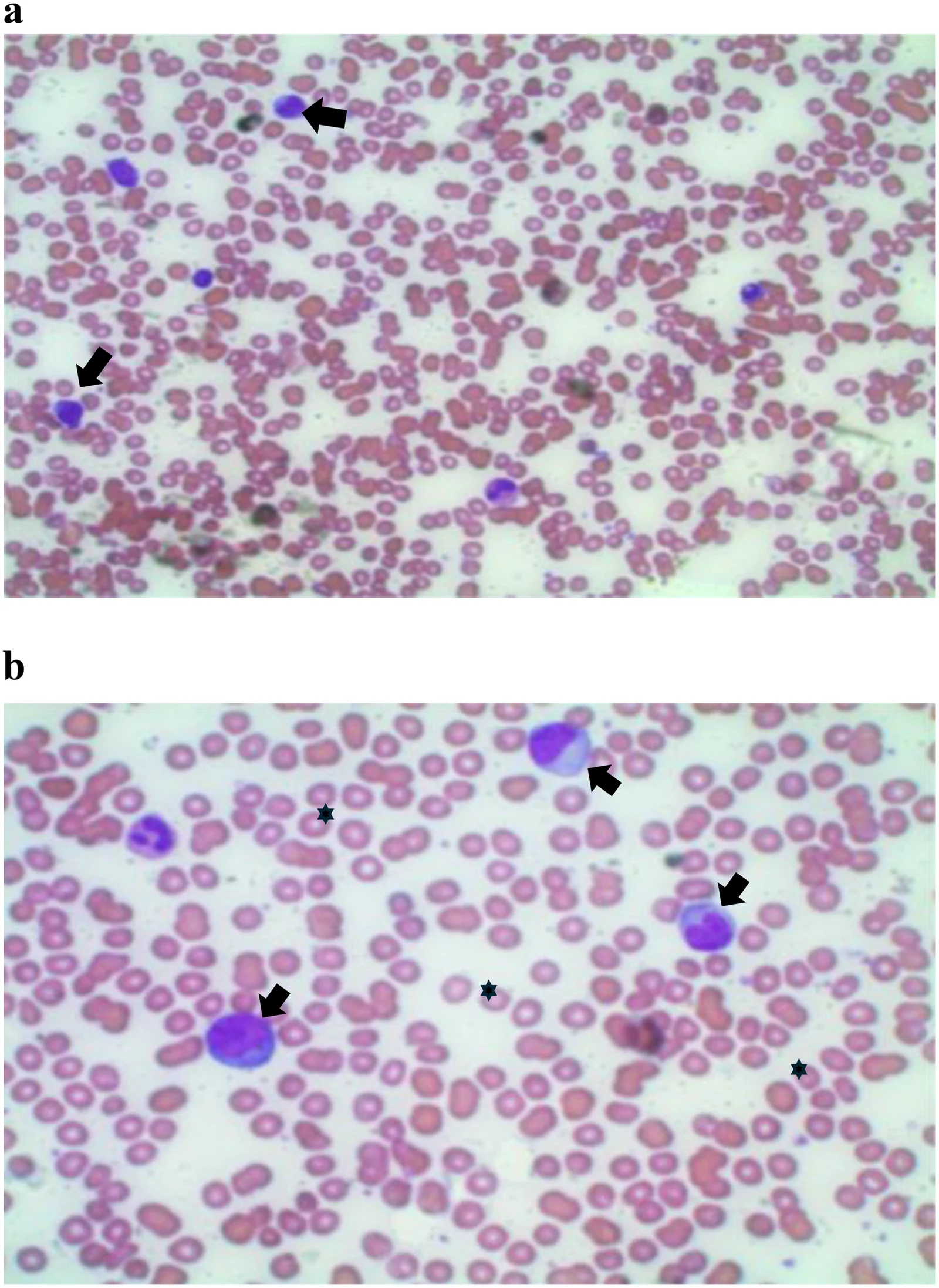
**a**: Peripheral smear revealing absolute lymphocytosis with numerous reactive lymphocytes (black arrow). **b**: Peripheral smear demonstrating numerous reactive lymphocytes (black arrow). Erythrocytes (black asterisk) are normocytic and normochromic

Upon admission, laboratory studies (Table 1) revealed elevated liver enzymes: aspartate aminotransferase (AST) 276 IU/L (N: 3–31 IU/L), alanine aminotransferase (ALT) 358 IU/L (N: 19–25 IU/L), alkaline phosphatase 297 IU/L (N: 30–120 IU/L), and total bilirubin 6.3 mg/dL (N: 0.2–1.2 mg/dL), with a direct component of 4.50 mg/dL (N: 0.1–0.4 mg/dL) and indirect 1.90 mg/dL (N: 0.1–0.8 mg/dL). Serum haptoglobin was < 10 mg/dL (N: 33–278 mg/dL) and lactate dehydrogenase was elevated at 925 U/L (N: 120–246 U/L). A complete blood count (CBC) (Table 2) demonstrated a mildly elevated white blood cell count (WBC) at 12,300/mm³ (N: 3.8–10.4 × 10^3^/mm³) with 58% lymphocytes. Hemoglobin was 8.0 g/dL (N: 12.0–16.0 g/dL) and hematocrit was 23.0% (35–47%), with an elevated reticulocyte count of 2.53% (N: 0.6–2.1%). A hepatitis panel (Table 3) was negative, but testing for Epstein-Barr virus (EBV) revealed positive EBV viral capsid antigen (VCA) IgG and a positive direct antiglobulin test was also noted. A peripheral blood smear (Fig. 1) demonstrated absolute lymphocytosis with reactive lymphocytes, 22% neutrophils, 74% lymphocytes, and 4% monocytes. Repeat EBV testing confirmed the presence of EBV VCA IgM, positive EBV VCA IgG, negative EBV nuclear IgG, and positive EBV early antigen IgG antibodies. A right upper quadrant ultrasound showed splenomegaly but no evidence of gallstones or biliary ductal dilation. Given the serologic and supportive radiologic findings, a diagnosis of acute primary EBV infection was made.

**Table 1:** Laboratory evaluation demonstrating a mixed Hepatocellular-cholestatic liver injury in acute Epstein-Barr virus infection

Laboratory Study	Result	Normal Range
Aspartate aminotransferase (AST)	276 IU/L	8–33 IU/L
Alanine aminotransferase (ALT)	358 IU/L	4–36 IU/L
Alkaline phosphatase	297 IU/L	44–147 IU/L
Total bilirubin	6.3 mg/dL	0.1–1.2 mg/dL
Direct bilirubin	4.50 mg/dL	0–0.3 mg/dL
Indirect bilirubin Lactate dehydrogenase Haptoglobin	1.90 mg/dL 925 U/L < 10 mg/dL	0.2–0.8 mg/dL 120–246 U/L 33–278 mg/dL

Note: laboratory findings at presentation demonstrating a mixed pattern of liver injury in acute Epstein-Barr virus infection

**Table 2:** Complete blood count (CBC) demonstrating anemia likely secondary to autoimmune hemolytic anemia (AIHA) in acute Epstein-Barr virus infection

White blood cell (WBC) count Red blood cell (RBC) count Hemoglobin Hematocrit Reticulocyte count	12,300/mm³ 2.5 × 10(6)/mcL 8.0 g/dL 23% 2.53%	4,500–11,000/mm³ x10(6)/mcL 12.0–16.0 g/dL 35–47% 0.6–2.1%

**Table 3:** Serologic and laboratory findings consistent with acute Epstein–Barr virus infection

Laboratory Study	Result
Direct antiglobulin test (DAT)	Positive
Anti-EBV viral capsid antigen (VCA) IgM	Positive
Anti-EBV viral capsid antigen (VCA) IgG	Positive
Anti-EBV nuclear IgG	Negative
Anti-EBV early antigen IgG	Positive
Viral hepatitis panel	Negative

Note: serologic pattern (VCA IgM+, VCA IgG+, EA IgG+, EBNA IgG–) is consistent with acute primary Epstein-Barr virus infection. Hepatitis panel was negative, excluding viral hepatitis as an etiology of liver injury

Over the course of her hospitalization, her liver function markers (AST, ALT, alkaline phosphatase, and bilirubin) gradually decreased, and anemia improved, with concurrent improvement in scleral icterus and jaundice following supportive care.

## Discussion

EBV-related elevation of transaminases is widely cited in the literature. However, significant hyperbilirubinemia and overt jaundice associated with IM is rare, and their presence is generally considered indicative of more severe disease, particularly in adult patients. Interestingly, a case series by Paez et al. reported that patients with hyperbilirubinemia were less likely to exhibit classical features of IM, such as pharyngitis: an observation also noted in our patient ​ [4]​.

The pathophysiology of icteric hepatitis in IM is believed to result from an exaggerated immune response ​ [4]​. Patients with elevated bilirubin levels often exhibit increased lymphocyte counts, elevated serum IgM, and higher concentrations of β2-microglobulin ​ [4]​. The heightened immune response is thought to contribute to the downstream formation of autoantibodies targeting red blood cells (RBCs).

Cold-type autoimmune hemolytic anemia (AIHA) represents a rare but recognized complication of IM, triggered by IgM antibodies against EBV that cross-react with RBC antigens through molecular mimicry ​ [6]​. Activation of the classical complement cascade ensues, leading to C3b deposition on RBCs and subsequent hemolysis ​ [6]​. The resultant intravascular hemolysis contributes to elevated circulating free hemoglobin levels and clinically apparent jaundice ​ [6, 7]​. Diagnostic confirmation typically includes a positive direct antiglobulin test (DAT) showing C3b deposition without IgG, accompanied by laboratory findings of hemolysis such as elevated lactate dehydrogenase, indirect hyperbilirubinemia, low haptoglobin ​and compensatory reticulocytosis [6, 7]​. Accordingly, this patient demonstrated laboratory evidence suggestive of immune-mediated hemolysis, including positive DAT and developed clinically significant anemia. Given that autoimmune hemolysis generally results in predominantly indirect hyperbilirubinemia, the degree of concurrent direct hyperbilirubinemia observed in this case suggests a multifactorial etiology with involvement of both hemolysis and EBV-associated cholestatic hepatitis.

In more severe cases of EBV-associated icteric hepatitis, particularly when serum bilirubin levels exceed 6 mg/dL, the underlying mechanism appears multifactorial. High viral loads within T lymphocytes, immune-mediated hepatic injury and intrahepatic cholestasis are all implicated. Notably, patients with jaundice have been found to have significantly higher EBV loads in CD3+, CD4+, and CD8 + T cells compared to other cell types, highlighting the key role of T cells in the disease process ​ [8]​. This differs from milder cases of EBV infection, where B cells are the primary targets ​ [8]​. The infiltration of EBV-infected T cells into the liver contributes to hepatic inflammation and damage, as evidenced by histological findings that typically reveal lymphocytic, rather than hepatocytic, infection ​ [8]​. Additionally, a cholestatic pattern of liver injury, characterized by elevated bilirubin and alkaline phosphatase levels, may be present, and is associated with more severe dysfunction, with serum bilirubin levels reported as high as 47.5 mg/dL in extreme cases ​ [5]​.

It is important to consider several factors in our patient that may have contributed to the severity of her presentation. The postpartum period is characterized by significant immune modulation, which can alter the body’s response to infections such as EBV, potentially leading to more severe manifestations, including increased liver involvement ​ [4]​. Additionally, while cefuroxime is generally well-tolerated, antibiotics have been associated with liver enzyme elevations and cholestasis, which, though unlikely, cannot be entirely ruled out as a contributor to the observed liver dysfunction ​ [4, 5]​. Moderate alcohol consumption also poses a risk for hepatic stress and may exacerbate the liver injury seen in EBV-induced hepatitis ​ [9]​. These factors are not commonly documented as direct contributors to EBV-induced jaundice. However, they may have contributed to the severity of our patient’s presentation, while EBV remained the primary underlying etiology.

In the present case, the patient’s jaundice was most consistent with a multifactorial process, driven by EBV-associated hepatocellular liver injury with cholestatic features accounting for the elevation in direct bilirubin and alkaline phosphatase in the absence of biliary obstruction. Second, laboratory evidence of immune-mediated hemolysis likely contributed to the indirect hyperbilirubinemia. Together, these factors best explain the severity of jaundice observed in an otherwise minimally symptomatic patient. This case of EBV-related hyperbilirubinemia differs from prior reports by illustrating a patient presentation dominated by hepatic and hematologic abnormalities, rather than classic infectious mononucleosis features, which highlights a diagnostically challenging and underrecognized phenotype of EBV infection.

## Conclusion

We present the case of a previously healthy woman who developed the rare manifestations of EBV-related jaundice and hyperbilirubinemia while remaining minimally symptomatic. IM should remain on the differential diagnosis not only for patients with elevated transaminases but also in those presenting with jaundice and hyperbilirubinemia, even in the absence of classic symptoms. Awareness of this atypical presentation may help avoid unnecessary diagnostic testing and interventions, as EBV-associated hepatitis is typically self-limiting and resolves with supportive care alone.

## Data Availability

Data sharing is not applicable to this article as no datasets were generated or analyzed during the current study.

## Abbreviations

IM: Infectious mononucleosis
EBV: Ebstein-Barr virus
UTI: Urinary tract infection
AST: Aspartate aminotransferase
WBC: White blood cell count
VCA: Viral capsid antigen
DAT: Direct antiglobulin test
AIHA: Autoimmune hemolytic anemia

## References

[1] Dunmire SK, Verghese PS, Balfour HH. Primary Epstein-Barr virus infection. J Clin Virol. 2018;102:84–92. 10.1016/j.jcv.2018.03.001. 29525635

[2] Kang MJ, Kim TH, Lee JH, et al. Infectious mononucleosis hepatitis in young adults: two case reports. Korean J Intern Med. 2009;24:381–7. 10.3904/kjim.2009.24.4.381. PMC2784984 19949739

[3] Fuhrman SA, Tatum AH, Tatum GB, et al. Marked hyperbilirubinemia in infectious mononucleosis: analysis of laboratory data in seven patients. Arch Intern Med. 1987;147:850–3. 10.1001/archinte.1987.00370050046008. 3034177

[4] Páez-Guillán EM, Campos-Franco J, Alende R, et al. Jaundice in relation to immune activation during Epstein-Barr virus-induced infectious mononucleosis. Am J Med Sci. 2023;365:270–8. 10.1016/j.amjms.2022.12.004. 36526005

[5] Shaukat A, Tsai HT, Rutherford R, Anania FA. Epstein-Barr virus induced hepatitis: an important cause of cholestasis. Hepatol Res. 2005;33:24–6. 10.1016/j.hepres.2005.06.005. 16112900

[6] Meloni DF, Pani A, Massidda M, et al. Hemolytic anemia linked to Epstein–Barr virus infectious mononucleosis: a systematic review of the literature. J Clin Med. 2025;14:1283. 10.3390/jcm14041283. PMC11856085 40004813

[7] Teijido J, Tillotson K, Liu JM. A rare presentation of Epstein-Barr virus infection. J Emerg Med. 2020;58:e71–3. 10.1016/j.jemermed.2019.11.043. 31973958

[8] Hara S, Tsurumi T, Sugimoto K, et al. Association of virus infected-T cell in severe hepatitis caused by primary Epstein-Barr virus infection. J Clin Virol. 2006;35:250–6. 10.1016/j.jcv.2005.07.009. 16181807

[9] Balasubramanian S, Kowdley KV. Effect of alcohol on viral hepatitis and other forms of liver dysfunction. Clin Liver Dis. 2005;9(1):83–101. 10.1016/j.cld.2004.10.004. 15763231

